# Osteoligamentous injuries of the medial ankle joint

**DOI:** 10.1007/s00068-015-0548-2

**Published:** 2015-07-04

**Authors:** P. Lötscher, T. H. Lang, L. Zwicky, B. Hintermann, M. Knupp

**Affiliations:** Department of Orthopaedic Surgery, Kantonsspital Baselland, 4410 Liestal, Switzerland

**Keywords:** Ankle sprain, Medial ankle ligaments, Deltoid ligament injury, Ankle fracture

## Abstract

Injuries of the ankle joint have a high incidence in daily life and sports, thus, playing an important socioeconomic role. Therefore, proper diagnosis and adequate treatment are mandatory. While most of the ligament injuries around the ankle joint are treated conservatively, great controversy exists on how to treat deltoid ligament injuries in ankle fractures. Missed injuries and inadequate treatment of the medial ankle lead to inferior outcome with instability, progressive deformity, and ankle joint osteoarthritis.

## Introduction

Ankle injuries are among the most common reasons for emergency department consultations. For a long time, diagnosis and therapy of ankle injuries and/or pain focused on the lateral side of the ankle. However, involvement of the medial side in sprains [1] and ankle fractures [2] is more frequent than believed. For example, in ankle sprains, the eversion type injuries have been linked to medial ankle instability [1].

Despite the growing awareness of the importance to identify osteoligamentous injuries of the medial ankle joint, great controversy exists on how to treat these patients. In deltoid ligament insufficiency, surgical reconstruction has become popular for patients who have failed previous conservative treatment. In ankle fractures, most authors recommend to stabilise fractures of the medial malleolus, however, usually do not reconstruct the medial ligaments unless fracture reduction is not possible.

The aim of this article is to provide an overview of the different entities and treatment concepts of osteoligamentous injuries of the ankle joint.

## Anatomy of the deltoid ligament and injury mechanism

The deltoid ligament complex spreads fan-shaped over the medial part of the ankle joint. It plays an essential role regarding stability against valgus and rotatory forces. The deltoid ligament consists of six distinct components, four superficial and two deep ligaments. The superficial ligaments (tibiospring ligament TSL, tibionavicular ligament TNL, superficial posterior tibiotalar ligament STTL and tibiocalcaneal ligament TCL) cross both the ankle and the subtalar joint, while the deep components (deep posterior tibiotalar ligament PTTL and anterior tibiotalar ligament ATTL) only cross the ankle joint [3]. The broad insertion of the superficial deltoid ligament on the spring ligament also plays a key role in the stabilising function of the medial ligaments. The superficial layers of the deltoid ligament particularly limit the talar abduction, while the deep layers limit the external rotation [4]. Both deep and superficial layers are equally effective in limiting pronation of the talus. Therefore the main causes of isolated deltoid ligament lesions are pronation or external rotation movements of the hindfoot.

## Clinical importance

In the following paragraphs, isolated injuries to the deltoid ligament and the role of the deltoid ligament in ankle fractures will be discussed.

### Acute and chronic injuries of the deltoid ligament

Isolated deltoid ligament injuries account for about 3–4 % of all ankle ligament injuries [1]. A large majority can successfully be treated conservatively. This usually involves cast immobilisation for 4–6 weeks with weight bearing as tolerated. Left untreated, they may lead to ongoing pain, instability and even progressive valgus deformity of the hindfoot. This is particularly the case in patients where the spring ligament has also been injured. Therefore, surgical reconstruction should be considered in combined injuries of the deltoid ligament and the spring ligament with or without involvement of the tibialis posterior tendon.

### Deltoid ligament injuries and ankle fractures

Ankle fractures often are a combination of bony and ligamentous injuries [5, 6]. It has been described that the deltoid ligament is involved in up to 40 % of ankle fractures [7]. About 80 % of the ankle fractures occur due to supination-external rotation (SER) injuries. In these ankles, a deltoid tear or a fracture of the medial malleolus is observed in all stage 4 fractures (according to the Lauge Hansen classification). In pronation-external rotation fractures involving the fibula, there is always a fracture of the medial malleolus or a deltoid ligament tear. These two fracture types are always unstable, and it is recommended to undergo open reduction and internal fixation in these cases.

#### Should we repair the deltoid ligament in ankle fractures?

Ankle fractures in combination with an osteoligamentous involvement of the medial ankle usually present as unstable injuries. Therefore, surgical treatment is often recommended.

Fibular fractures are treated with plate fixation; however, the treatment of a concomitant deltoid ligament injury is discussed controversially. Many authors suggest that the deltoid ligament does not need to be repaired, if anatomical reduction of the fibula is possible [8–13].

In patients with medial malleolar fractures (SER stage 4), about a quarter also have an associated disruption of the deep deltoid ligament [14]. Therefore, fixation of a medial malleolar fracture with only a screw or plate without addressing the injured deltoid ligament may not restore ankle joint stability. Tornetta showed that 26 % of all patients with a fixed medial malleolar fracture had an evident incompetence of the deltoid ligament, seen radiographically [15]. Thus, intraoperative stress exams and radiographs are recommended to determine if medial ankle stability has been restored (Fig. 3). If the medial clear space remains wide after fixation of the malleolar fracture, repair of the deltoid ligament is recommended.

#### Outcome of medial malleolar fractures versus deltoid ligament injuries

Stufkens et al. [16] compared the clinical outcome of SER-4 fractures at 13-year follow-up in two groups of patients: (a) those patients with an intact deltoid ligament but medial malleolar fracture (*n* = 19) and (b) patients with a partial or complete rupture of the deltoid ligament with an intact medial malleolus (*n* = 17). Lateral and—if present—medial malleolar fractures were surgically treated with plate fixation of the fibula and screw fixation of the medial malleolus. They showed that SER-4 fractures with a fracture of the medial malleolus had a poorer outcome (lower AOFAS hindfoot score) than those with a partial or complete rupture of the deltoid ligament. Furthermore, arthroscopic assessment showed a significant higher risk for loose intraarticular bodies in the group of the medial malleolar fracture with an intact deltoid ligament. Several other authors point out the possibility that these cartilage lesions have a significant influence on the long-term outcome of patients with medial malleolar fractures [2, 17–21].

## Diagnosis of medial ankle injuries

**Fig. 1:**
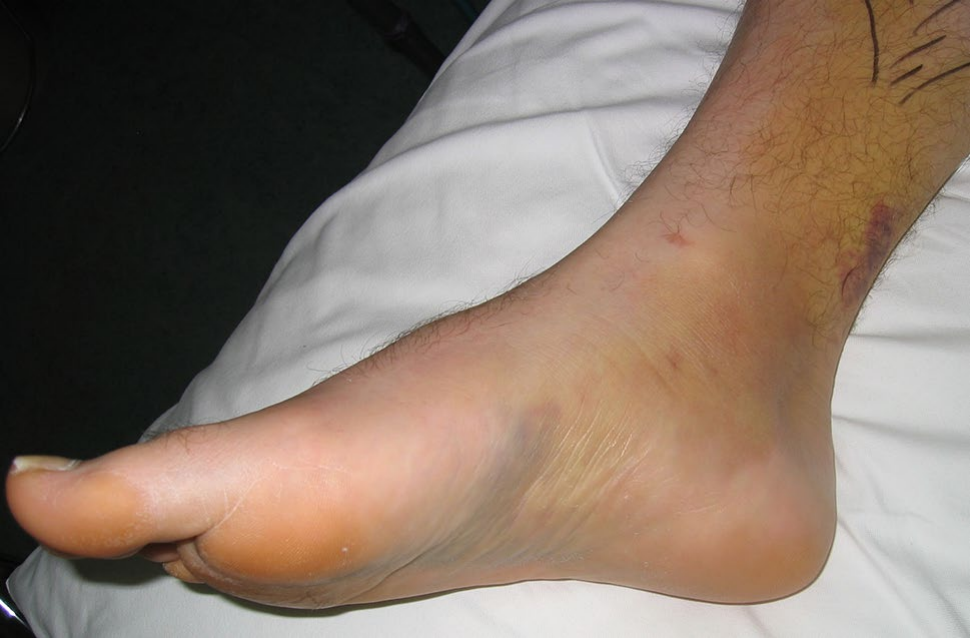
Acute deltoid ligament injury. Patients usually present with ecchymosis, swelling, and tenderness along the medial part of the ankle joint. Weight bearing may be impossible due to pain and instability

The diagnosis of medial ankle ligament injuries is based on patients’ medical history and clinical findings. Patients with an acute injury of the deltoid ligament usually complain of pain in the anteromedial part of the ankle joint and give a history of either an eversion-pronation trauma or a supination-external-rotation trauma. Generally, ecchymosis and tenderness along the deltoid ligament are present (Fig. 1). Furthermore, loading of the ankle joint is critical and associated with a feeling of instability.

**Fig. 2:**
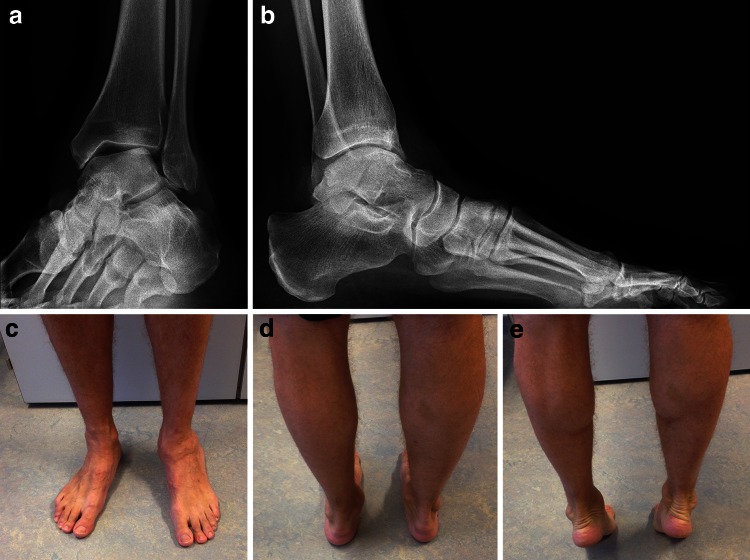
Chronic medial ligament instability. Radiographical (**a**, **b**) and clinical (**c**–**e**) distinct medial instability with talar tilt and hindfoot valgus of the *left foot*. The lateral X-ray (**b**) shows an intact talonavicular joint with the talar head still in an articulating position, suggesting that the spring ligament is intact. While standing on tiptoe (**e**), hindfoot valgus disappears due to the intact and functioning posterior tibial muscle

In patients with chronic medial ligament instability, accurate diagnosis may be more demanding. These patients usually report a medial “giving-way”, especially while walking down a hill or stairs. A hallmark in getting the diagnosis is the tenderness at the medial gutter of the ankle joint [22]. Not only the injured ligaments themselves but also the synovitis of the medial part of the ankle joint are responsible for this anteromedial pain. The drawer test and the talar tilt test may be positive. Clinically the patient may present with a flatfoot with prominence of the medial malleolus, pronounced hindfoot valgus and pronation of the affected foot. In contrast to patients with a tibialis posterior tendon dysfunction, the patients are able to actively correct the hindfoot valgus deformity and perform a single heel rise (Fig. 2).

Standard radiographs are used to exclude fractures after acute trauma. Preoperative stress radiographs are not recommended because of the lack of additional information and the potential to further damage the injured structures. In the chronic medial ankle instability, standard weight-bearing radiographs are taken to assess segmental deformities in all three planes. MRI may help to identify a weakening or avulsion of the medial malleolus, osteochondral lesions, affection of the spring ligament and the tibialis posterior tendon. However, MRI has been shown to be clearly less reliable in detecting ligamentous deficits compared to arthroscopic assessment [23]. Furthermore, MRI has been shown to be unhelpful for determining whether operative or conservative treatment of the common SER-type ankle fractures is necessary [24].

## Intraoperative diagnostic measures

**Fig. 3:**
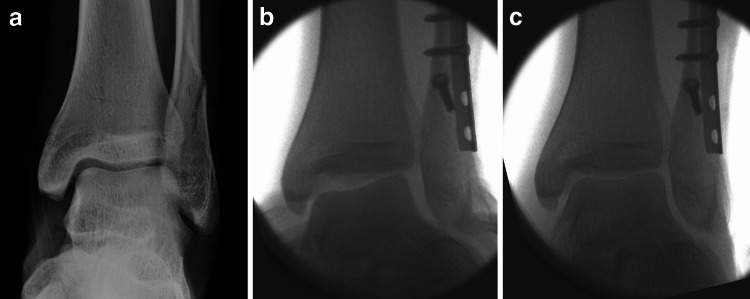
SER-4 fracture: combination of a spiral fracture of the fibula and a deltoid ligament injury (**a**). After anatomical fracture reduction of the fibula, the medial clear space remains wide in the talar tilt stress view (**b**). Stable condition after deltoid ligament reconstruction (**c**)

If surgical treatment is necessary, the diagnosis can be completed using intraoperative fluoroscopy and arthroscopy with the patient under anaesthesia. Intraoperative stress radiographs may allow assessing syndesmotic instability and/or opening of the medial clear space while performing valgus stress in the ankle mortise (Fig. 3). Additionally, clinical tests like the talar tilt and the anterior drawer test can be performed to gain clinical information about the pattern of injury.

### Arthroscopy

Ankle arthroscopy allows assessing the degree and pattern of ankle instability of both the medial and lateral side. Generally, injuries to the deltoid ligament occur at the proximal insertion site, and its insertion zone at the medial malleolus shows a naked area of periosteum where the ligament is detached. Furthermore, associated cartilage lesions can be identified.

The following manoeuvres allow the assessment of the instability:

- “axial traction” to quantify the amount of opening of the tibiotalar space and to test the possibility of being able to insert a 5 mm arthroscope into the tibiotalar joint space
- “talar anterior draw” to assess the medial and anteromedial instability
- “valgus stress” to detect laxity/instability of the medial ligaments
- “varus stress” to detect laxity/instability of the lateral ligaments

Ankle arthroscopy may also allow treating associated lesions such as removal of loose bodies, debridement, microfracturing, and intraoperative control of fracture reduction.

## Treatment

### Conservative treatment

Acute isolated deltoid injuries are usually immobilised in a cast for 6 weeks with gradual return to the pre-injury activities. In chronic insufficiency, physical therapy such as muscular strengthening, proprioceptive training and coordination training is initiated. Orthotics with a medial support, bracing or taping may additionally be used to provide mechanical support and enhance proprioception through skin pressure.

Ankle fractures with deltoid ligament injuries usually are unstable fractures requiring open reduction and internal fixation. In conservative treatment, immobilisation and partial weight bearing are recommended.

### Surgical treatment

Surgical reconstruction of deltoid ligament injuries is indicated if open reduction internal fixation in ankle fractures is not possible due to medial soft tissue interposition and in cases where chronic deltoid insufficiency is present. Relative indications include unstable ankle fractures, ankle fractures with syndesmotic injuries and acute ruptures in professional athletes.

#### Surgical technique

The surgical technique varies, depending on the extent and location of the ligament injury: (a) injuries at the proximal part of the deltoid (type-I lesions), (b) injuries at the intermediate part of the deltoid (type-II lesions), and (c) injuries at the distal part of the deltoid and spring ligaments (type-III lesions) [25].

A slightly curved incision, 4–8 cm in length, is made, starting 1–2 cm proximal to the medial malleolar tip and headed towards the medial aspect of the navicular bone. After the dissection of the fascia, the deltoid ligament and the posterior tibial tendon are exposed.

In Type-I lesions, the insertion area at the anterior aspect of the medial malleolus is exposed. These lesions typically originate in the interval area, the small fibrous septum between the tibiocalcaneal and tibiospring ligaments. The insertion area at the anterior border of the medial malleolus is roughened, and a suture anchor or a transosseous suture is placed 4–6 mm above the tip (e.g. anterior colliculus) of the medial malleolus. The detached ligament is taken by the suture and the open interval is firmly closed.

In Type-II lesions, the incompetent and typically hypertrophic ligament is divided into two flaps. The deep part, which has its origin at the navicular tuberosity, is fixed to the medial malleolus as it is done when treating a proximal lesion, using a bony anchor. The superficial part, which has its origin at the medial malleolus, is fixed distally to the superior edge of the navicular tuberosity using another bony anchor.

In Type-III lesions, a bony anchor is used to fix the detached deltoid and spring ligaments to the navicular tuberosity. If the remaining tissue of the spring ligament is of bad quality, the distal part of the posterior tibial tendon is used to augment the ligament reconstruction.

In patients where the ankle instability persists and ligament quality is insufficient (less than 5 %), direct reconstruction with anchors may not be possible. In these cases, autologous reconstruction using a free tendon graft (e.g. plantaris tendon graft) should be considered. The graft is passed through two 3.2 mm drill holes 2–8 mm above the medial malleolar tip and through another dorso-plantar drill hole in the navicular bone. Holding the foot in a neutral position, the graft is fixed with absorbable sutures under slight tension. Attention has to be paid to reconstruct the tendon in a strict anatomical position and to not over tighten the ligament construct.

## Results

### Isolated ligament repair

In a series of 52 patients with a superficial deltoid ligament insufficiency, Hintermann et al. found a type-I lesion of the superficial deltoid ligament in 71 %, a type-II lesion in 10 % and a type-III lesion in 19 % of the cases [22]. Repair of the deltoid ligament was performed in all 52 cases as described previously; it was necessary to repair the spring ligament in 24 % and the lateral ligaments in 77 %. The clinical results in this series were considered as “good to excellent” in 90 %, “fair” in 8 %, and “poor” in 2 %. This appears to show that the management of deltoid ligament injuries as described previously, leads to favourable results.

## Discussion

Acute injuries of the ankle joint play an important socioeconomic role accounting for up to 25 % of the injuries treated in medical practice [26] and up to 30 % of all sports injuries [27–29]. However, deltoid ligament injuries are a frequently missed diagnosis, both in the acute injuries and chronic symptomatic patient. Failure to recognise osteoligamentous injuries of the medial ankle and inappropriate treatment may lead to disabling sequalae and eventually to degenerative joint disease.

Most patients with acute deltoid ligament injuries can be treated conservatively with immobilisation in a plaster for 6 weeks. If conservative treatment has failed and patients report chronic instability or recurrent ankle sprains, surgical reconstruction of the insufficient deltoid ligament is recommended. Reconstruction in the acute setting should be considered in combined injuries involving the medial and the lateral ligaments or the spring ligament.

Arthroscopic assessment of 288 acute ankle fractures revealed injury to the medial ligaments more frequently than clinically expected (39.6 %) [2]. Decision making for the treatment of ankle fractures requires knowledge of the stability of the fracture pattern: in the most common Weber B fractures (supination-external rotation ankle fractures), the decision for operative or nonoperative treatment is based on the stability of the ankle. Unstable fractures usually have a better outcome if they are managed operatively [30]. Instability of the medial ankle can either result from a deltoid ligament tear or a fracture of the medial malleolus. Non-recognising medial instability in ankle fractures has been shown to negatively influence the outcome; therefore, the surgeon needs to be aware of the state of the medial soft tissue when deciding which treatment modality of the ankle fracture is undertaken.

Whether the medial soft tissues need to be reconstructed or not has been discussed controversially. A majority of the authors recommend deltoid reconstruction, only if the torn ligament does not allow for adequate fracture reduction. However, if the ankle remains unstable after fracture reduction and fixation, reconstruction of the ligaments is necessary. This is particularly true in patients with a concomitant fracture of the medial malleolus and a deltoid ligament tear (Fig. 3)

The type of the medial instability greatly influences the outcome of the fracture. Both, Tejwani et al. [31] and Stufkens et al. [16], found better outcome in patients who presented with a tear of the deltoid ligament when comparing them to patients with a fracture of the medial malleolus. They evaluated functional outcomes of bimalleolar and “bimalleolar-equivalent” (disrupted deltoid ligament, intact medial malleolus) fractures in 266 patients and 36 patients, respectively. Even if the deltoid ligament injury was not repaired, the group of patients with deltoid injuries had a significant better outcome compared to the group of patients with medial malleolar fractures. The reason for this observation is thought to be the accompanying cartilage injury in the patients with a medial malleolus fracture [2, 17–21].

Recognition of the importance of medial ankle stability has given reason to question the need for syndesmotic stabilisation in ankle fractures. It is a matter of debate whether or not, in cases with a stable medial ankle, the distal tibiofibular joint should be fixed. Furthermore, Jones and Nunley recently demonstrated that bimalleolar-equivalent fractures fixed with lateral fixation of the fibula and deltoid ligament repair had a comparable subjective, functional and radiological outcome when compared to fixation of the fibula with a syndesmotic repair [32]. This observation questions whether syndesmotic screw fixation is needed in cases where the deltoid ligament is repaired or in ankle fractures with intact medial ankle ligaments.

## Summary

Deltoid ligament injuries in ankle fractures and sprains have a higher incidence than clinically believed and are frequently missed.

Acute, isolated deltoid tears are treated conservatively. In case of involvement of the spring ligament, surgery is considered. In ankle fractures, surgical repair of the deltoid is recommended if the ankle remains unstable after reconstruction or if reduction of the fracture is not possible due to soft tissue interposition.

Further studies are needed to answer the question whether fixation of the distal tibiofibular joint in ankles with preserved medial stability is required and if reconstruction of the deltoid ligament may replace lag screw/tight rope fixation in syndesmotic injuries.
